# Peripheral (deep) but not periventricular MRI white matter hyperintensities are increased in clinical vascular dementia compared to Alzheimer's disease

**DOI:** 10.1002/brb3.438

**Published:** 2016-02-16

**Authors:** Charles D. Smith, Eleanor S. Johnson, Linda J. Van Eldik, Gregory A. Jicha, Frederick A. Schmitt, Peter T. Nelson, Richard J. Kryscio, Ronan R. Murphy, Clinton V. Wellnitz

**Affiliations:** ^1^ Department of NeurologyUniversity of Kentucky College of MedicineLexingtonKentucky; ^2^ Magnetic Resonance Imaging and Spectroscopy CenterUniversity of KentuckyLexingtonKentucky; ^3^ Alzheimers Disease CenterSanders‐Brown Center on AgingUniversity of KentuckyLexingtonKentucky; ^4^ Department of Anatomy and NeurobiologyUniversity of Kentucky College of MedicineLexingtonKentucky; ^5^ Department of Pathology & Laboratory MedicineUniversity of Kentucky College of MedicineLexingtonKentucky; ^6^ Department of StatisticsUniversity of KentuckyLexingtonKentucky; ^7^ Department of Diagnostic RadiologyMayo ClinicScottsdaleArizona

**Keywords:** Image quantitation, magnetic resonance imaging, vascular dementia, white matter ratings

## Abstract

**Background and purpose:**

Vascular dementia (VAD) is a complex diagnosis at times difficult to distinguish from Alzheimer's disease (AD). MRI scans often show white matter hyperintensities (WMH) in both conditions. WMH increase with age, and both VAD and AD are associated with aging, thus presenting an attribution conundrum. In this study, we sought to show whether the amount of WMH in deep white matter (dWMH), versus periventricular white matter (PVH), would aid in the distinction between VAD and AD, independent of age.

**Methods:**

Blinded semiquantitative ratings of WMH validated by objective quantitation of WMH volume from standardized MRI image acquisitions. PVH and dWMH were rated separately and independently by two different examiners using the Scheltens scale. Receiver operator characteristic (ROC) curves were generated using logistic regression to assess classification of VAD (13 patients) versus AD (129 patients). Clinical diagnoses were made in a specialty memory disorders clinic.

**Results:**

Using PVH rating alone, overall classification (area under the ROC curve, AUC) was 75%, due only to the difference in age between VAD and AD patients in our study and not PVH. In contrast, dWMH rating produced 86% classification accuracy with no independent contribution from age. A global Longstreth rating that combines dWMH and PVH gave an 88% AUC.

**Conclusions:**

Increased dWMH indicate a higher likelihood of VAD versus AD. Assessment of dWMH on MRI scans using Scheltens and Longstreth scales may aid the clinician in distinguishing the two conditions.

## Introduction

White matter hyperintensities (WMH) on magnetic resonance imaging (MRI) have a heterogeneous underlying neuropathology, variable genetics, and complex relationship with clinical symptoms and associated copathologies (Drayer 1988; Kertesz et al. 1990; Debette and Markus 2010). Patients with Alzheimer's disease (AD) and vascular dementia (VaD), although they may both demonstrate WMH on MRI scans, may have differing relationships between WMH and clinical symptoms leading to diagnosis. WMH on MRI may serve as a marker for more widespread white matter microstructural alterations measurable using nonstandard MRI techniques (Maillard et al. 2014; Maniega et al. 2015; Tuladhar et al. 2015a; Wang et al. 2015a).

**Figure 1:**
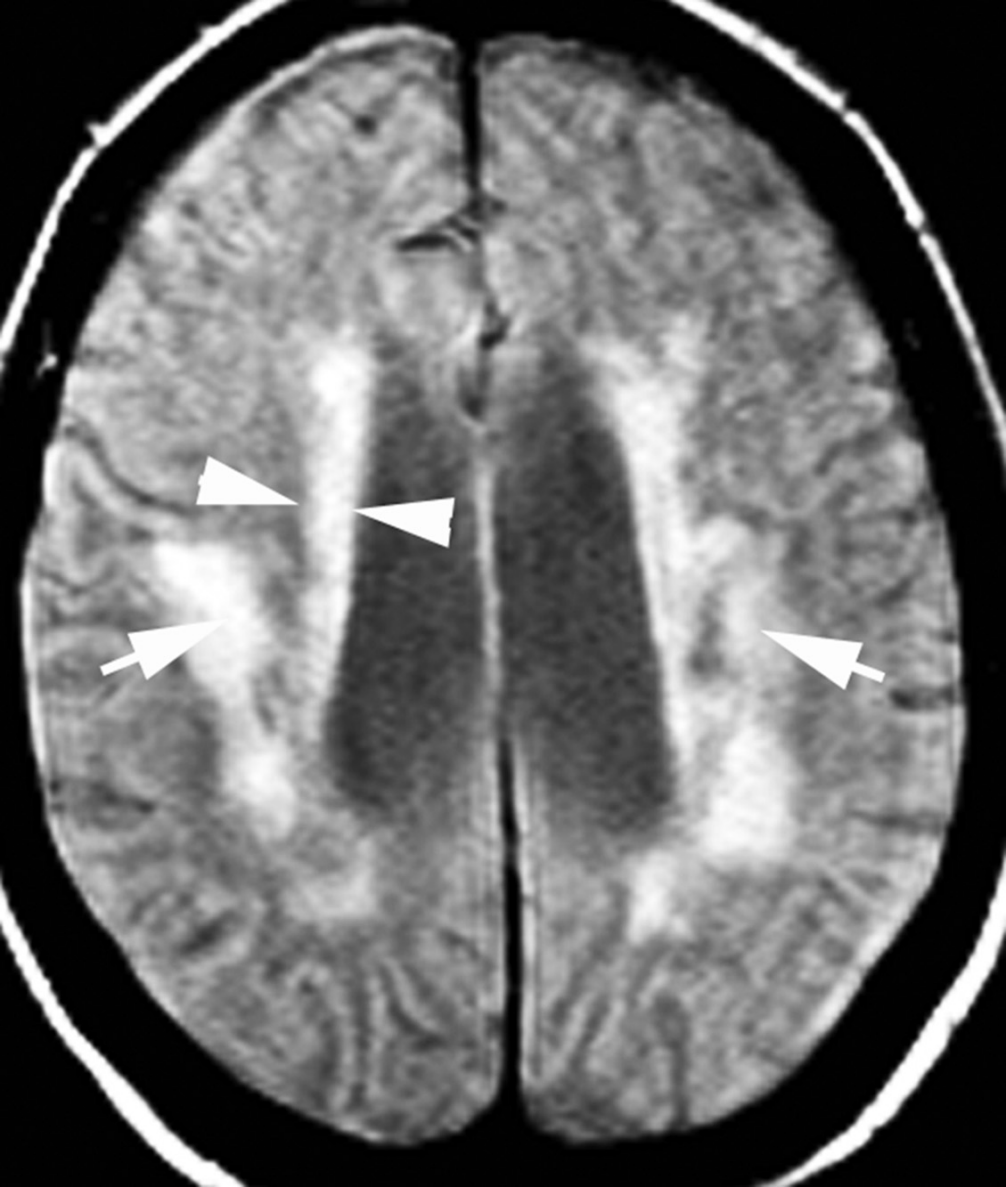
Illustration of dWMH versus PVH. PVH are indicated by paired facing arrowheads on the right; dWMH are indicated by arrow.

Variable patterns of WMH distribution have been associated with specific risk factors, such as stroke, alcohol abuse, and tobacco use (Rostrup et al. 2012). Deep WMH (dWMH) refers to hyperintensities in the centrum semiovale and other deep white matter extending up to the subcortical U‐fibers, in contrast to hyperintensities immediately bordering the lateral ventricles, termed periventricular WMH (PVH; Fig. 1). Increased dWMH versus PVH have been found in normal community‐dwelling persons with lower scores on tests of executive function (Soriano‐Raya et al. 2012), in patients with significant hypertension (Firbank et al. 2007) and in patients with depression (Krishnan et al. 2006). Patients with diabetes demonstrate association of slowed processing speed with dWMH, whereas PVH are associated with reduced attention and executive function (Tiehuis et al. 2008). Thus, there is evidence for a potentially useful distinction between deep and periventricular WMH.

In this study we examined the relationship between dWMH and PVH in patients clinically diagnosed with either AD or VaD. We performed independent semiquantitative ratings of WMH, and quantified WMH volume to validate these ratings. We found that dWMH, in contrast to PVH, predicted the diagnosis of VaD, indicating that dWMH are a substrate of more profound impairment than PVH.

## Methods

### Participant characteristics

We collected the MRI scans from 210 subjects who underwent evaluation in an outpatient specialist memory disorders clinic at the University of Kentucky Alzheimers Disease Center (ADC) in a 40‐month time window as described previously in detail (Schmitt et al. 2012). Patients were referred from the community for the evaluation because of a memory complaint or suspected dementia, typically suspected AD. Presence of a caregiver was required to provide a reliable history. Each patient underwent a full neurologic and medical examination, and had laboratory testing including hemogram, electrolytes, liver and renal function tests, thyroid function studies, serum B12 and folate levels, and other laboratories as deemed appropriate. After evaluation, including consideration of the MRI scan formal report, patients were given a diagnosis and prescribed treatment. Patients provided consent (together with a caregiver or relative) under University of Kentucky Medical Institutional Review Board approved procedures.

Diagnosis of possible and probable AD used NIA‐ADRC criteria, and possible or probable vascular dementia (VaD) was based on the extended Hachinski Ischemic Scale (Lau et al. 1988) and DSM‐IV (American Psychiatric Association, 2000) criteria. Current terminology now includes VaD under the rubric “Vascular cognitive impairment or dementia” (VCID), but we retained the VaD designation because subjects were diagnosed as such under DSM‐IV. Frontotemporal dementia, dementia with Lewy bodies, corticobasal degeneration, multisystem atrophy, and normal pressure hydrocephalus (NPH) were diagnosed according to published literature standards. Mild cognitive impairment used “age associated memory impairment” criteria current at the time of study (Crook and Larrabee 1991), prior to the Peterson criteria era.

Two patients without a recorded diagnosis had large‐vessel infarcts by MRI (one old left anterior cerebral artery, one acute right parietal infarct). One patient with an old right frontal infarct was diagnosed possible AD, but excluded because of this confounding pathology. An additional patient diagnosed as possible VAD harbored a large right ventricular cyst and was thus excluded due to the presence of this potentially cofounding pathology. Exclusions are summarized in Table 1. After exclusions, 142 subjects remained in the group for study, 129 with AD diagnosis, and 13 with VaD.

**Table 1:** Number of Subjects excluded for diagnoses other than Vascular dementia or Alzheimer's disease

Diagnosis	Excluded subjects (technical excludes)
Dementia NOS	10 (1)
MCI	8 (2)
Metabolic	2 (0)
VaD^a^	3 (3)
MSA‐P	1 (0)
AD^a^	10 (10)
CBD	2 (0)
DLB	2 (0)
FTD	5 (1)
NPH	1 (0)
Not Demented	3 (1)
No Diagnosis	19 (2)
Total	68 (20)

The number of subjects excluded because of technical problems with the scans (excessive motion, missing images or variation from standard protocol) is given in parentheses. Patients with AD or VaD diagnosis were only excluded for poor technical quality of the scans.

CBD, corticobasal ganglionic dementia; DLB, dementia with Lewy bodies; FTD, frontotemporal dementia; MCI, mild cognitive impairment; MSA‐P, multisystem atrophy, parkinsonian type; NOS, not otherwise specified; NPH, normal pressure hydrocephalus; VaD, vascular dementia; AD, Alzheimer's disease.

a Two/Three VaD excluded due to poor scan quality, 1/3 excluded because of a large right ventricular cyst treated as a confound.

### Scan protocol

A standardized MRI imaging protocol was performed on clinical Siemens 1.5 T magnetic resonance imaging scanners according to a dementia‐specific sequence series. This series included a sagittal scout, axial T1‐weighted, T2‐weighted, and proton density (PD) images, and two T1‐weighted coronal series, the first in a standard orientation and a second series oblique oriented parallel to the main axis of the hippocampus. For segmentation and analysis of WMH, the following sequences were used: Axial images, 0.898 × 0.898 × 5 mm, TR 2600, TE 27 ms (PD) and TE 81 ms (T2‐weighted), and T1‐weighted with TR 610 ms, and TE 14 ms. All series consisted of 19 slices with a 1.5 mm interslice gap.

### Image processing and rating & validation

**Figure 2:**
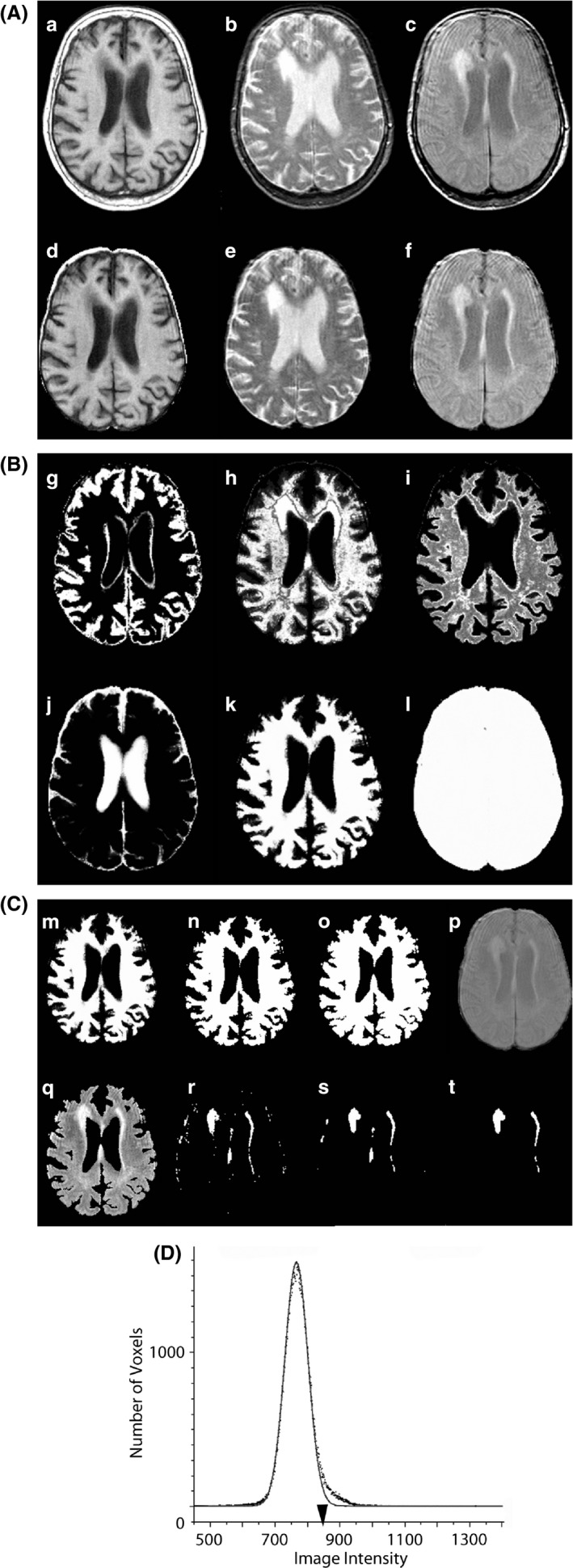
Processing steps for WMH volume measurement. (A) Top row: (left to right, a–c), original T1‐, T2‐, and PD‐weighted images; Bottom row (d–f): corresponding skull‐stripped, N3‐corrected images. (B) Segmented images. Top row (left to right, g–i): gray‐ and white‐matter (WM) segmentations; two tissues were modeled for WM because of the signal differences between normal‐appearing WM and WM hyperintensities. Bottom row: CSF(j), total WM (sum of h+i), and sum of GM(g), WM(k), and CSF(j) images(l). (C) Masking. Top row (left to right): succession of WM masks beginning with WM segmented image, followed by thresholded binary WM image, and dilated binary WM image. Rightmost image is skull‐stripped PD image. Bottom row: PD image masked by dilated WM binary, masked image thresholded at 2.33 S.D., gaussian‐filtered image, final edited WMH image for quantitation. (D) Histogram demonstrating gaussian fit and threshold value (black arrowhead). Note shoulder in the histogram on the right representing WMH plus tail of WM gaussian pixel distribution.

PD and T2 images were affine registered and resliced into the native images space of the T1 image in SPM8 (http://www.fil.ion.ucl.ac.uk/spm/). The T1 image was skull‐stripped using the FSL version 5.07 Brain Extraction Tool (BET v2.1) with robust center estimation (http://fsl.fmrib.ox.ac.uk/fsl). The binary extraction mask isolated the brain portion of the PD and T2 images by multiplication. These images were then field‐corrected using the N3 algorithm (Fig. 2A). Residual skull signal was removed during the segmentation step.

Continuing in SPM8, the New Segment protocol was followed with the skull‐stripped, N3‐corrected T1 and T2 images entered into a multimodal segmentation with component gray matter (GM), white matter (WM), and cerebrospinal fluid (CSF). The PD image did not improve segmentation. A template created from a separate group of 146 similar‐aged normal subjects was applied during segmentation.

Component GM, WM, and CSF images are scaled to intensity values between zero and one. Total component volumes were estimated by thresholding each component image at 0.333, and summing the voxel volumes. Importantly in this method, WM was modeled in New Segment as two separate tissues, each incorporating two gaussians. The sum of these tissues gave more reliable total WM segmentation than when a single tissue alone represented WM (Fig. 2B).

The segmented WM image was converted to a binary mask with a threshold of 0.333, and dilated once in 2.5 days to create a WM mask (MIPAV version 7.1.1 morphology; http://mipav.cit.nih.gov/). The dilation step expanded the outline of the binary mask to capture WMH at the WM – CSF boundary that may have been misclassified in the segmentation due to partial volume effects.

Next the skull‐stripped, registered, N3‐corrected PD image was multiplied by the WM mask to remove CSF and GM voxels (Fig. 2C). The histogram of this WM‐extracted PD image was fit with a gaussian profile to estimate a mean and standard deviation (MIPAV version 7.1.1). A *P*‐value of 0.01 was chosen as the threshold to define WMH (2.33 × SD) relative to the WM voxel mean in each individual image (Fig. 2D). The thresholded PD image was smoothed with a resolution normalized (*z* = 1) gaussian kernel filter to remove noise pixels. Hand‐editing was often necessary to remove artifacts in the remaining image, particularly at the base of the brain at the level of the large blood vessels. The total volume of hyperintensities exceeding the threshold was recorded for each subject as total WMH volume.

In order to compare images at similar contrast, ratings on the Scheltens, Fazekas, and Longstreth scales were made on PD images in a darkened room with the display window center set at the histogram mean, and window width set at ten times the standard deviation. The Scheletens and Fazekas scales require separate scoring for dWMH and PVH. PVH represent bands and caps that follow the contour of the lateral ventricles with variable thickness and degrees of irregularity. Deep WMH extend from the immediate periventricular region of PVH through the centrum semiovale and other regions of deep white matter to the subcortical U‐fibers. Scheltens PVH are scored on a 0–6 scale and dWMH on a 24 point scale, guided by explicit definitions for size and number of hyperintenstities in each region. We used this scale as our primary visual rating because of its specificity and scale range. Fazekas ratings capture global aspects of dWMH and PVH, each on a 0–3 point scale. The Longstreth scale rates density, confluence, and extent of WMH on a 0–9 range without explicitly distinguishing PVH from dWMH.

### Rating scales and validation

Two independent observers followed the same protocol described to gain an estimate of repeatability and reliability of WMH measures in 34 subjects. Interobserver measurement of WMH showed a linear correlation coefficient adjusted *r* ^2^ of 0.95, with a regression slope of 0.98, and offset (intercept) of 190 voxels. Nonparametric rank correlations between observers for WMH volume and WM rating scales are shown in Table 2A. Reliability was greatest for the WMH volume protocol, followed in order by Longstreth rating, Scheltens deep WMH rating, and Scheltens PVH rating (Scheltens et al. 1993) (all correlation *P*‐values < 0.0001). In our hands the Fazekas ratings were less reliable than for Scheltens. Observer 2 Fazekas ratings were less reliable relative to total WMH volume than observer 1's. Validity of the WMH volume measurement protocol was assessed by correlating WMH volume with both WMH rating scales (Table 2B), demonstrating satisfactory reliability on all scales.

**Table 2:** (A) Spearman rank correlations between WMH volume and WMH rating scales from two independent observers in 34 subjects, as a validation of the reliability of the WMH measurement protocol. (B) Rank correlations between WMH volume and WMH rating scales for observer 1, who performed measurements on all 142 subjects

Observer 2	WMH volume	Longstreth	Scheltens PVH	Scheltens deep WMH	Fazekas PVH	Fazekas deep WMH
(A)						
WMH Volume	0.93 <0.0001	0.96 <0.0001	0.49 0.003	0.36 0.03	0.54 0.01	0.58 0.004
Longstreth	0.94 <0.0001	0.82 <0.0001	0.69 <0.0001	0.64 <0.0001	0.59 <0.0001	0.69 <0.0001
Scheltens PVH	0.59 0.0002	0.72 <0.0001	0.65 <0.0001	0.45 0.007	0.28 0.19	0.58 0.005
Scheltens Deep WMH	0.44 0.009	0.68 <0.0001	0.44 0.009	0.77 <0.0001	0.50 0.02	0.64 0.001
Fazekas PVH	0.45 0.03	0.61 0.002	0.38 0.08	0.38 0.08	0.36 0.10	0.59 0.004
Fazekas Deep WMH	0.45 0.03	0.54 0.01	0.34 0.12	0.48 0.02	0.27 0.23	0.67 0.0006

Observer 1	Longstreth	Scheltens PVH	Scheltens Deep WMH	Fazekas PVH	Fazekas Deep WMH
(B)					
WMH Volume	0.83 <0.0001	0.72 <0.0001	0.60 <0.0001	0.55 <0.0001	0.71 <0.0001

WMH, white matter hyperintensities; PVH, periventricular white matter.

*P*‐value for correlation given under coefficient.

These comparisons show strong correlations, but do not imply a direct numerical correspondence between observers on any of the WMH measures. Rather, the comparisons show that the measures increase together strongly and coherently. They also show that increasing WMH volume corresponds to what clinicians see as increased WMH on T2‐weighted images, for example, using the Longstreth scale.

### Statistical methods

Comparisons of means used two sample *t*‐tests; comparison of proportions used chi‐square tests; comparison of Hachinski scores used a Wilcoxon rank sum test (JMP version 9). For analysis of WMH, logistic regression models were calculated incorporating log‐transformed ratings as the independent variables, and age at MRI scan, gender, education, whole‐brain volume in cubic centimeters, MMSE, and Hachinski score as adjustment variables, with diagnosis (VaD vs. AD) as the outcome. Effect testing used the likelihood ratio; a *P*‐value of 0.05 or less was considered significant.

To normalize distributions, all measures of WMH were scaled by adding 0.5 (to avoid zero) and then by taking the natural logarithm of the result (log normal transform). Log‐normalized scales were treated as continuous variables. Measures of WMH were added to separate models as follows: (1) Two Scheltens scales for Caps and Bands (range 0–6), and for white matter hyperintensities (range 0–24 (Scheltens et al. 1993)), (2) Longstreth rating of WMH (Longstreth et al. 1996) (range 0–9), and (3) WMH volume. Receiver operator characteristic (ROC) curves and tables were computed from each of the three models.

## Results

Demographic and testing variables are shown in Table 3. Only mean age at MRI scan differed between VaD and AD patients; the VaD patients were approximately 5 years older. Nine percent of the patients had VaD, the others were diagnosed with AD.

**Table 3:** Demographic and testing data. VaD patients were older than the AD patients on average, and had higher Hachinski scores

Diagnosis (n)	Age, years	Male/Female (n)	Education, years	MMSE (0–30)	Hachinski (Mod 0–13)
AD (129)	75.1 ± 7.6	42/89	12.5 ± 4.0	19.4 ± 4.9	0 [0–1]
VaD (13)	80.5 ± 6.4	5/8	13.4 ± 4.1	19.3 ± 5.4	3 [1–5.5]
Compared	*t* = 2.5 *P* = 0.01	X^2^ = 0.22 *P* = 0.7	*t* = 0.81 *P* = 0.42	*t* = 0.00 *P* = 0.95	X^2^ = 13.7 *P* = 0.0002

AD, Alzheimer's disease; VaD, vascular dementia; MMSE, mini‐mental status examination score (range 0–30).

X^2^ = ChiSquare; Hachinski vascular dementia score, median [IQR], Wilcoxon Test.

Logistic regression incorporating two Scheltens scales demonstrates that only the deep WMH rating was significantly related to the diagnosis of VaD (Table 4). Adjustment variables reaching significance were education in the Longstreth regression (LR chi‐square 4.5, *P* = 0.03), and, as expected from its relationship with the diagnosis of VaD, the Hachinski score (*P*‐values between 0.002 and 0.0001). Periventricular hyperintensity rating was not significant. Both the Longstreth rating and WMH volume were significantly related to the diagnosis of VaD, but neither of these measures distinguished deep from periventricular hyperintensity.

**Table 4:** Results of logistic regression model for WMH using standard ratings of PVH and dWMH (first and second rows; Scheltens, Fazekas), and total WMH (third and fourth rows; Longstreth and WMH volume). Including an interaction term for age and WMH did not alter the result. We found a similar effect with both the Scheltens and Fazekas scales, despite the wider score range of the Scheltens scale

AD versus VaD	PVH	dWMH	Total WMH
Scheltens	LR 1.9 *P* = 0.17	LR 11.5 *P* = 0.0007	–
Fazekas	LR 0.23 *P* = 0.63	LR 11.6 *P* = 0.0006	–
Longstreth	–	–	LR 15.1 *P* = 0.0001
WMH Volume	–	–	LR 12.2 *P* = 0.0005

LR, Likelihood ratio chi‐square; WMH, white matter hyperintensities; PVH, periventricular white matter; dWMH, deep white matter; AD, Alzheimer's disease; VaD, vascular dementia.

**Figure 3:**
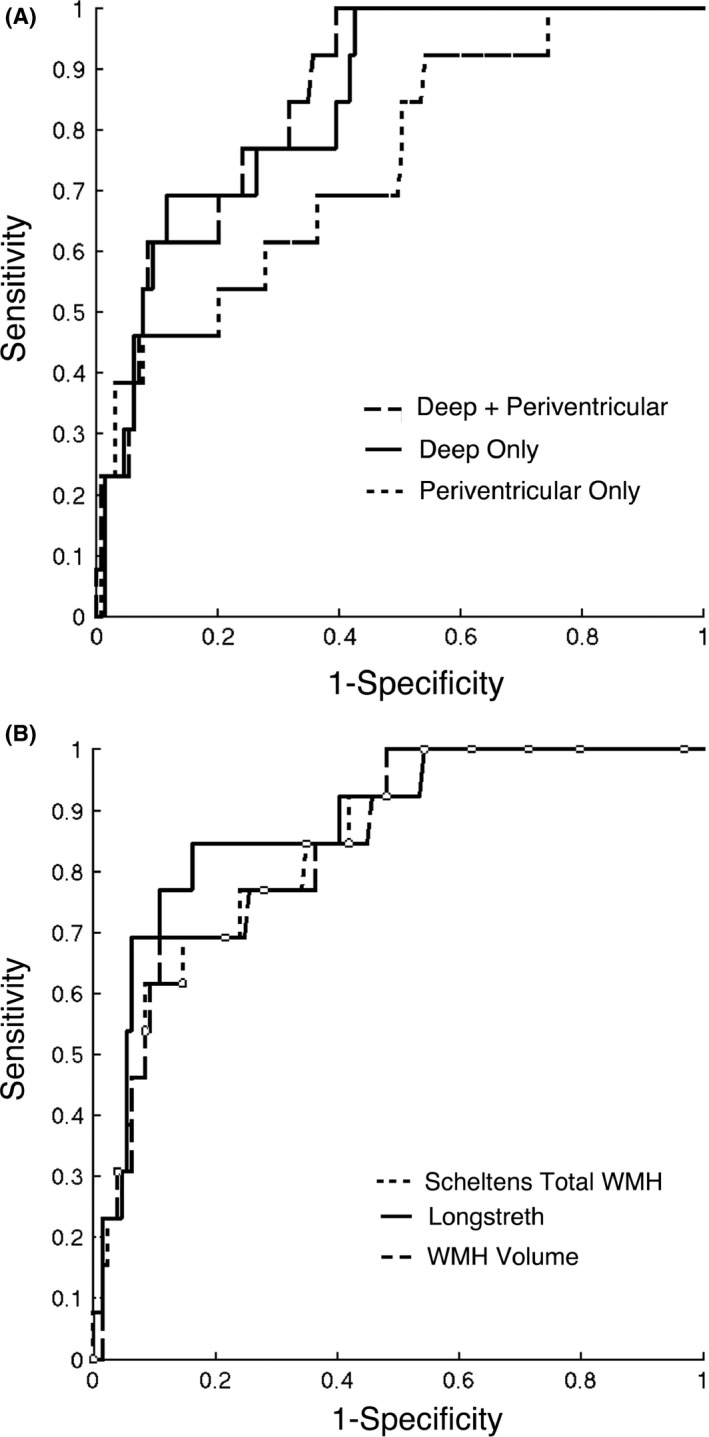
Receiver operator characteristic (ROC) curves for models described in Statistical Methods section. These curves indicate quality of the classification VAD versus AD; increased performance curves approach the left and upper borders of the graph. (A) Comparison between Scheltens dWMH and PVH ratings demonstrating improved classification of VAD using dWMH (dashed line) compared to PVH (dotted line), (B) Comparison of overall WMH rating methods. Scheltens (dashed line) and WMH volume (dotted line) show similar classification performance, slightly improved with Longstreth (solid line).

The ROC curves for Scheltens ratings (area under the curve (AUC) 86%) and total white matter volume (AUC 85%) were essentially identical (Fig. 3). The ROC curve for Fazekas ratings was similar (AUC 87%, not shown). The Longstreth rating gave a slightly better overall performance in predicting VaD versus AD (Fig. 3B, solid line; AUC 88%). At optimum, the Longstreth scale had an 85% sensitivity and 68% specificity for VaD. In contrast, the model without any WMH measures had an AUC of 71%, with optimum of 54% sensitivity and 48% specificity, close to chance. This weak prediction was related only to the increased age of our VaD patients previously noted in Table 3.

## Discussion

The most important finding of our study was that deep white matter hyperintensities (dWMH) better distinguish VaD from AD than periventricular hyperintensities (PVH). Two different semiquantitative rating scales that separately rate dWMH and PVH on MRI brain images, Scheletens and Fazekas, gave similar results. We interpret these finding to mean that clinicians, in evaluating standard clinical FLAIR and T2‐weighted MRI studies of the brain, can sharpen assessment of VaD by increasing focus on dWMH in addition to other vascular findings such as lacunar infarcts, microbleeds, and focal encephalomalacia (Noh et al. 2014). The simplest explanation is that larger amounts of dWMH are associated with increasing disruption of brain networks concerned with executive function, gait stability, and urinary control. Recent studies have shown that specific tracts crossing regions of WMH may show corresponding expected functional consequences (Jacobs et al. 2012; Birdsill et al. 2014; Duering et al. 2014; Tuladhar et al. 2015b).

We also found that increased scores on a global rating scale, Longstreth (Longstreth et al. 1996), was associated with VaD. There was a very strong relationship between objectively measured total white matter hyperintensity volume (PVH plus dWMH) and Longstreth rating, supporting the validity of that semiquantitative observer‐based scale. The Longstreth scale was developed as a tool to assess effects of vascular disease on the brain, and rates both PVH and dWMH together, under the assumption that PVH and deep WMH extents increase together. While this is generally true, there appears to be value in separating PVH and dWMH in the evaluation of VaD.

We emphasize that the scans were performed on routinely available clinical scanners using a standard clinical imaging protocol. Although we performed quantitation of WMH under a rigorous image processing protocol in this study, this was used mainly for validation. In addition, the protocol can be implemented using publicly available image processing programs without using special proprietary scripts (available in detail from the first author). Moreover, it is not necessary to use these programs, as we showed strong correlation between WMH rating scores and WMH volume. The relationship was logarithmic, and showed that increased WMH volume is strongly predictive of what a clinician means by increased WMH when evaluating a scan, consistent with other studies (Gouw et al. 2006, 2008a). Thus, for aiding diagnosis of VaD, semiquantitative ratings are sufficient.

The common form of white matter hyperintensities may comprise two types: a type associated with vascular injury to small vessels, due to long‐standing hypertension for example, and a second type of uncertain etiology related to aging (Fazekas et al. 1998; Smith et al. 2000a,b). Distinguishing between the two may be difficult because vessel damage from cerebrovascular disease increases with duration of disease (e.g., age), and the aging‐associated type may involve, at least partially, a vascular component distinct from atheroscerosis or hypertensive injury (arteriolosclerosis).

Periventricular white matter abnormalities particularly are age‐related and likely due to alterations in cerebrospinal fluid dynamics at the ependymal lining of the ventricles, in some instances associated with ventricular enlargement. Deep white matter hyperintensities are pathologically heterogeneous, in some cases consisting of focally widened spaces lined by atrophic myelin centered on fibrohyalinized vessels, and in others larger areas of gliosis, myelin rarefaction, and fiber loss consistent with ischemia (Gouw et al. 2011; Schmidt et al. 2011). The first kind of dWMH appears as scattered punctate foci on MRI have no proven clinical significance. Deep white matter dilated perivascular spaces, recently associated with altered cerebrospinal fluid drainage dynamics (Weller et al. 2015), should not be confused with these punctate foci.

The second dWMH kind is vascular in nature (Young et al. 2008), most likely to increase over time, and associated with clinical evidence of executive dysfunction, gait difficulties, and urinary abnormalities (Maillard et al. 2012; Chutinet and Rost 2014; Allan et al. 2015). The midlife CAIDE dementia risk score, that includes hypertension, obesity, cholesterol, and physical activity factors, predicts WMH volume 20 years later (Vuorinen et al. 2015), although in cross‐sectional studies these factors are better predictors of large artery disease than WMH (Wardlaw et al. 2014). Diabetes is recognized as another cause of microvascular pathology (Nelson et al. 2009). Increased WMH are associated with decreased gray matter and CSF volumes (Zi et al. 2014), but the contribution of cardiovascular risk factors to this relationship has been questioned recently (Wang et al. 2014; Arvanitakis et al. 2015).

Heterogeneity in genetic influence on WMH has also recently been demonstrated by the finding that heritability was significantly higher in hypertensive (0.41) versus nonhypertensive (0.13) individuals in a 2243 patient GWAS study (Adib‐Samii et al. 2015). Single‐nucleotide polymorphisms (SNPs) predicting WMH were different between hypertensive and nonhypertensive patients (Adib‐Samii et al. 2015; Haffner et al. 2015). So far the only genetic locus clearly identified as associated with WMH is 17q25; the salient gene or genes within this locus remains unknown (Fornage et al. 2011). The presence of apolipoprotein epsilon‐4 allele (ApoE4), a known strong genetic risk factor for AD, strengthened the relationship between vascular risk factors and increased WMH. Although controversial (Brickman et al. 2014, 2015), symptomatic noncarriers have shown greater WMH than ApoE4 carriers, perhaps because WMH then become a stronger determinant of cognitive alterations (Morgen et al. 2015).

Other modifiers of WMH may include comorbid pathologies such as AD, where increased WMH has been found in some studies (Gouw et al. 2008b; Chui and Ramirez‐Gomez 2015). In AD, frontal cortical thinning has been associated with WMH and executive dysfunction (Ye et al. 2015). Alterations in myelin integrity found in AD may be secondary to axonal fiber loss or injury (Radanovic et al. 2013). Presence of these AD alterations may confer vulnerability of the axon‐myelin unit to specific non‐AD injury types (Erten‐Lyons et al. 2013; Kim et al. 2015), reflected genetically as different arrays of SNPs predicting WMH and suggesting synergisms (Chao et al. 2013; Yoon et al. 2013; Kester et al. 2014). However, in a recent longitudinal study of cognition in baseline normal subjects, amyloid burden‐defined AD pathology and MRI‐defined vascular pathology appeared to represent independent processes, with only additive, not multiplicative, effects on rate of cognitive decline, a conclusion supported by others (Lo and Jagust 2012; Barnes et al. 2013; Haight et al. 2013; Vemuri et al. 2015).

We interpret our results to mean that increased dWMH are more likely to be associated with a diagnosis of VaD than AD. A strong caveat to this interpretation is that the images were known to the clinicians making the diagnosis, and therefore the finding could represent a “self‐fulfilling prophecy” since WMH are evident on the scans. There are two reasons we think this is not the case, although we cannot entirely exclude it. First, clinicians used radiologist reports of scans rather than performing semiquantitative ratings or WMH volume measurements on the scans themselves. As scans on AD patients are often also reported as showing “periventricular white matter ischemic lesions”, matters of extent or deep versus periventricular pattern are not detailed in these reports. Second, Hachinski scores were increased in the VaD group. Clinical features are embedded in the Hachinski scale, not scan findings, including subcortical pattern of impairment, presence of hypertension or stroke, and characterization of progression. AD patients had a Hachinski score median of zero, clearly different from the VaD patients (median 3). The interquartile range overlapped between the AD and VaD group at one, suggesting the groups were quite different on clinical findings underlying the diagnosis.

One explanation for the unique contribution of dWMH to VaD diagnosis is the increased potential for the disruption of multiple functionally important networks supporting cognition (Medaglia et al. 2015). Decoupling of functional and structural connectivity by WMH has been demonstrated in recent studies (Reijmer et al. 2015; Wang et al. 2015b). Local functional regions tend to be densely connected, with these dense sets of nodes linked by sparse long‐range connections (Park and Friston 2013). An association has been shown between clinical depression and dWMH in the superior longitudinal fasciculus within the frontal lobe, suggesting that dWMH could disrupt critical long‐range integrative pathways (Sheline et al. 2008). While global WMH burden has been related to diminished network connectivity and impaired cognition (Pinter et al. 2015; Tuladhar et al. 2016), much further work is needed to relate regional WMH within specific tracts and the functional and cognitive consequences thereof.

Caution in the interpretation of our results is in order. Our patients were seen in a memory disorders clinic where the frequency of AD was high, and it is in this setting that our findings are most relevant. Patients with large‐vessel stroke were rare in this clinic and for that reason we could not address the issue of VaD subtypes involving such strokes. We did not have pathologic confirmation that small vessel disease was the predominant pathology explaining dementia, thus our interpretation remains on clinical grounds. Our model for WMH adjusted for differences in age, gender, education, whole brain volume, MMSE, and Hachinski score. Nonetheless, it should be kept in mind that the number of patients with VaD was relatively small.

In summary, we suggest clinicians focus on dWMH as a clue to vascular contributions to cognitive impairment and to rate their scans as part of clinical data collection, whether explicitly as dWMH versus PVH (Fazekas, Scheltens) or with emphasis on dWMH as part of a global WMH rating such as Longstreth. The way we now think of dWMH is that they reflect not a surrogate measure of vascular risk, but rather the impact of vascular risk factors on deep white matter. In some cases, we observe extensive dWMH in the absence of canonical vascular risk, nonetheless indicating impact, but from as yet unknown, perhaps genetic, influences.

## Conflict of Interest

The authors have no potential financial conflicts of interest to disclose.
